# Therapeutic drug monitoring in oncology - What’s out there: A bibliometric evaluation on the topic

**DOI:** 10.3389/fonc.2022.959741

**Published:** 2022-11-10

**Authors:** Jana Stojanova, Jane E. Carland, Bridin Murnion, Vincent Seah, Jim Siderov, Florian Lemaitre

**Affiliations:** ^1^ Department of Clinical Pharmacology and Toxicology, St. Vincent’s Hospital, Sydney, NSW, Australia; ^2^ Interdisciplinary Centre for Health Studies (CIESAL), Universidad de Valparaiíso, Valparaiíso, Chile; ^3^ School of Clinical Medicine, Faculty of Medicine and Health, Sydney, NSW, Australia; ^4^ Faculty of Medicine and Health, University of Sydney, Sydney, NSW, Australia; ^5^ Pharmacy Department, Austin Health, Heidelberg, VIC, Australia; ^6^ Université de Rennes, CHU Rennes, Inserm, EHESP, Irset (Institut de recherche en santé, environnement et travail), Rennes, France; ^7^ INSERM, Centre d’Investigation Clinique, Rennes, France

**Keywords:** therapeutic drug monitoring, pharmacokinetic, antineoplastic agents, kinase inhibitors, bibliometric analysis

## Abstract

Pharmacological therapy is the mainstay of treatment for cancer patients. Despite wide interpatient variability in systemic drug concentrations for numerous antineoplastics, dosing based on body size remains the predominant approach. Therapeutic drug monitoring (TDM) is used for few antineoplastics in specific scenarios. We conducted a rapid bibliometric evaluation of TDM in oncology to capture a snapshot of research in this area over time and explore topics that reflect development in the field. Reports with the composite, indexed term ‘therapeutic drug monitoring’ in the title and abstract were extracted from MEDLINE (inception to August 2021). Reports related to applications in cancer were selected for inclusion and were tagged by study design, antineoplastic drugs and concepts related to TDM. We present a timeline from 1980 to the present indicating the year of first report of antineoplastic agents and key terms. The reports in our sample primarily reflected development and validation of analytical methods with few relating to clinical outcomes to support implementation. Our work emphasises evidence gaps that may contribute to poor uptake of TDM in oncology.

## Introduction

Therapeutic drug monitoring (TDM) is the practice of determining systemic drug concentrations to optimise patient outcomes, mainly by adjusting drug dosage. It has been shown to be of benefit for drugs with a narrow therapeutic window, significant inter- and intra-individual pharmacokinetic variability, an established concentration-effect relationship and where information about circulating drug concentrations is helpful for clinical management (1). Although TDM has been used to support dosing decisions for some antineoplastics since the 1970s (1), it is not universally applied. TDM guided dosing is accepted for specific antineoplastics in specific clinical contexts, including high-dose methotrexate, busulfan and thiopurines (2). However, dosing based on weight and/or body surface area (BSA) continues to be the dominant approach despite observation of wide interpatient variability in systemic drug concentrations (2).

While there are many aspects to consider when evaluating the suitability of concentration-based dosing in oncology, one issue is that exposure-response relationships are not systematically evaluated during regulatory approval. This is illustrated by an examination of US Food and Drug Administration’s clinical pharmacology reviews for biologicals used in oncology; of 15 agents registered between 2005 and 2016, only five had documented exposure-response relationships (3). Following commercialisation in the post-marketing authorisation setting, most exposure-response work is performed through academic initiatives. As a result, it is difficult to determine whether an individual therapeutic agent is a poor candidate for concentration-based dosing in a particular clinical context, or simply that evidence is lacking or of poor quality.

The recent boom in targeted oral and biological therapeutics has changed clinical practice in cancer therapy and has resulted in numerous novel agents entering the market annually. For the targeted oral agents, a single, maximally tolerated universal dose is typically marketed, however most of these drugs are substrates of metabolic enzymes and drug transporters. Of note, dosage adjustment is suggested for managing adverse drug reactions in the summary of product characteristics sheet for a number of these drugs, implicitly suggesting an exposure-response relationship; two examples are regorafenib and ibrutinib (4, 5). Large interpatient variability in systemic concentrations is increasingly recognised, provoking numerous initiatives to evaluate the suitability of concentration-based dosing for these agents (6, 7). While available targeted oral agents outnumber antineoplastic biologicals (both targeted agents and checkpoint inhibitors), the latter are increasingly used. These are similarly marketed at a single, maximally tolerated fixed dose. Although drug exposure is less likely to be impacted by a patient’s physiological characteristics, concentrations have been related to effect for some agents (8). For the most part, research initiatives are academic and the evidence base has unfolded according to the clinical need and specialist areas of particular research groups.

Both traditional and targeted agents are approved following clinical trials involving participants that are relatively homogenous in terms of age, body size and ethnicity, and a lower degree of complex comorbidities. It is therefore often through research initiatives after introduction of the drug to routine clinical practice that the impact of obesity, extremes in age, comorbidities and genetic differences, among other aspects, are evaluated.

We conducted a rapid bibliometric evaluation of the literature referring to the TDM of antineoplastic agents, to capture a snapshot of the research in this area over time and explore topics that reflect development in the field. We characterised studies by publication type to explore the degree to which clinical evaluations have been undertaken within the greater body of literature. We sought to evaluate the impact of technological developments and novel approaches, such as alternative sampling strategies, over time. We were also interested in the timing and frequency of reports for agents that involved special populations, where the application of concentration-based dosing may be particularly important.

## Methods

### Search strategy

Reports with the composite, indexed term ‘therapeutic drug monitoring’ in the title and abstract were extracted from MEDLINE, from inception to August 3, 2021. This was a rapid scoping exercise. A subset of reports that included specific terms related to the fields of oncology/haematology and a list of antineoplastic agents were extracted (Appendix 1).

### Selection criteria and screening

Studies were included if they reported on TDM in oncology. There was no restriction on the type of report or language, except for corrigendum or errata which were not included. Reports related to myeloablation for hematopoietic stem cell transplantation, or those referring to agents that have not reached global markets to date were excluded. Screening of titles and abstracts, and initial tagging, was performed using Rayyan by four reviewers (9). Discrepancies were discussed and resolved by the review team.

### Tagging of therapeutic agents and key concepts

Tagging for therapeutic agents and key concepts was performed in Microsoft Excel. A column was assigned to each term and the presence of the term in the title and/or abstract was indicated. Additional terms were identified through iterations of the process. Tagging of a term was nonspecific; for example, occasionally therapeutic agents were referred to in the abstract as an auxiliary agent, such as co-therapy or an internal standard, rather than the object of the report. We did not correct for such instances and accepted this limitation. Tagging of words with different spelling was performed individually, but reported together, for example, pediatric/paediatric or haematology/hematology. Tagging of words and their most common acronyms was performed individually, but reported together, for example tandem mass spectroscopy/’MS/MS’. For reports without an abstract (n=25), the full text was reviewed to identify the publication type and the antineoplastic agent that was the object of the report, however concept terms were not reviewed for these reports. We did not tag for type of malignancy.

### Categorization and grouping

Tags related to study design were grouped into four categories: assay development and validation; modelling and simulation; clinical trials and primary studies; and reviews and perspectives (Appendix 2). Pharmacological agents were grouped into categories: cytotoxic antineoplastics; kinase inhibitors; hormonal antineoplastics; monoclonal antibodies and other non-cytotoxic antineoplastics.

### Data analysis and presentation

Data summaries were developed in R version 4.12 (10). Time series figures were prepared using Prism 9 (GraphPad, San Diego, CA).

## Results

**Figure 1:**
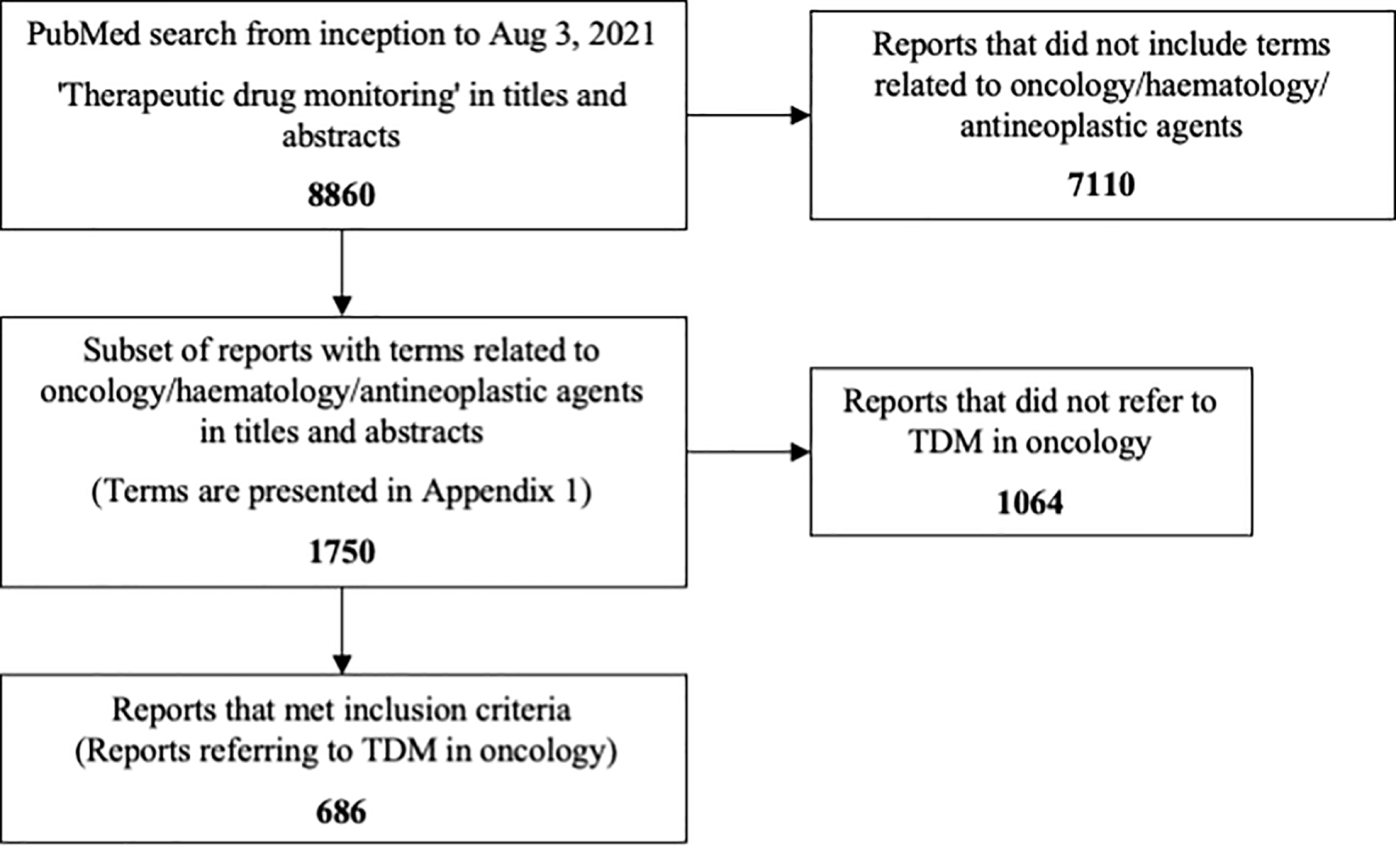
Flow chart presenting the study inclusion process.

There were 8860 reports with the term ‘therapeutic drug monitoring’ in titles and abstracts, and 1750 were identified for screening. Of the total set, 686 (7.7%) were included as referring to TDM in oncology (**Figure 1**). Reports were identified that related to 27 cytotoxic antineoplastics, 25 kinase inhibitors, 8 hormonal antineoplastics, 7 biological targeted agents and 3 other non-cytotoxic antineoplastics. Twenty-three reports (3%) did not refer to any specific neoplastic agent, most of which were general reviews or perspectives on the topic.

Most publications concerned analytical aspects (n=278, 40.5%); 23.9% were clinical or cost-effectiveness studies (n=164), 26.8% were reviews and perspectives (n=184) and 10.9% concerned modelling and simulation (n=72). Most clinical studies were observational: case reports (n=44), prospective cohort (n=41), retrospective cohort (n=35), case series (n=25); only 0.9% of all identified reports were randomized or non-randomized controlled trials where TDM was the intervention (n=6); these were conducted 2011-2021. Cost-effectiveness was the objective of 25 reports. Twelve reports were categorised into two of the four publication categories.

**Figure 2:**
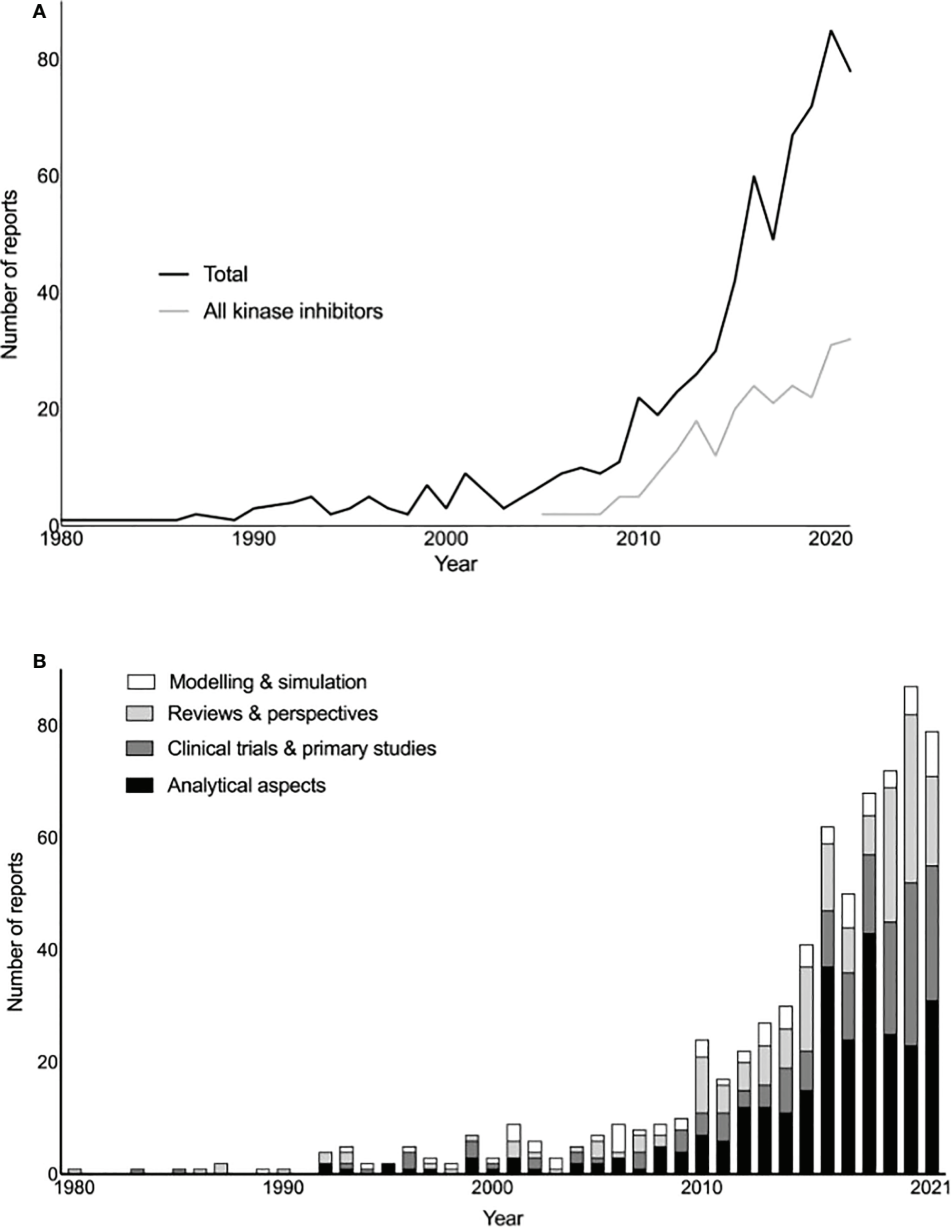
**(A)** Total reports and reports focusing on kinase inhibitors over time. **(B)** Total reports based on publication type over time.

The first report identified was published in 1980 and concerned methotrexate (**Figure 2A**). A relatively low publication rate on the topic of TDM in oncology (1-20 per year) was observed from 1980 until 2008 when the rate increased, aligning with the appearance of reports on kinase inhibitors, which were the object of 35.3% of all identified reports (n=242) (**Figure 2A**). Between 1980 and 1990 most publications identified were reviews and perspectives, while publications focusing on analytical aspects or clinical/other primary studies gradually increased in frequency from 1990 (**Figure 2B**).

**Figure 3:**
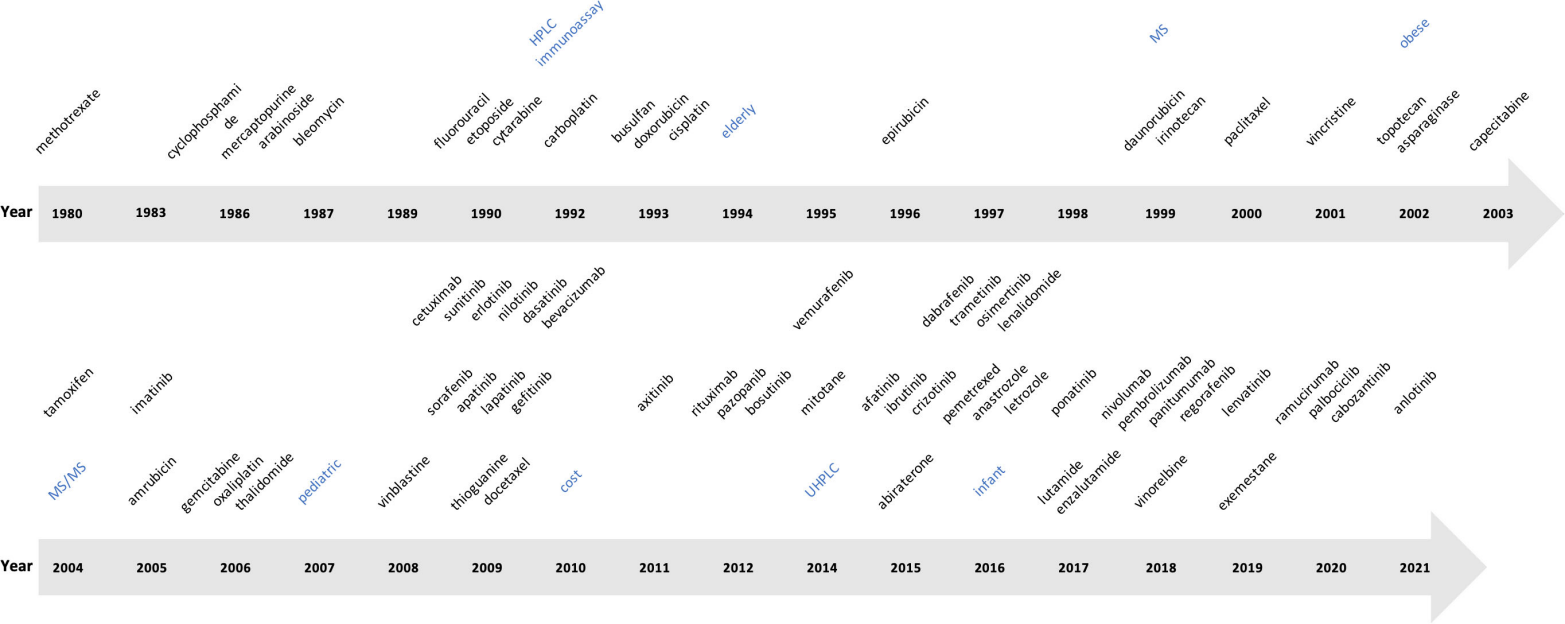
Timeline of the year of first report identified for individual antineoplastic agents and selected concept terms (blue). HPLC: high-performance liquid chromatography; MS: mass spectrometry; MS/MS: tandem mass spectrometry; UHPLC: ultra-high-performance liquid chromatography.

Most cytotoxic antineoplastic drug classes had agents with first reports identified in the 1980s or 1990s; first reports for agents from the taxane and vinca alkaloid classes were identified in 2000 (paclitaxel) and 2001 (vincristine), respectively (**Figure 3**). The first report of agents from the hormonal antineoplastic class was identified in 2004 (tamoxifen), although reports for most other agents in this class were first identified in 2014 (mitotane) and later. The first report for a kinase inhibitor was identified in 2005 (imatinib), with multiple reports covering a variety of agents identified from 2009 onwards. The first report covering antineoplastic antibodies was identified in 2009 (cetuximab) (**Figure 3**).

When focusing on the most frequently reported antineoplastics, the proportion of the different publication types varied by agent (**Table 1**). For example, asparaginase was the object of a higher number of clinical studies (48%) and reviews (36%), relative to reports on analytical aspects (12%). In contrast, most publications relating to kinase inhibitors focused on analytical aspects, for example, 76% of the 33 publications identified for nilotinib. Similarly, reports about analytical aspects accounted for at least 40% of reports for methotrexate and taxanes. The proportion of reports about modelling and simulation was typically 10% or less, with the exception of platinum agents (26%).

**Table 1:** Publication type identified for the most commonly reported cytotoxic and non-cytotoxic antineoplastics (25 reports or more).

DRUG	Total	Analytical method development and validation	Clinical trials and primary studies	Modelling and simulation	Reviews and perspectives
**Cytotoxic antineoplastics**					
methotrexate	78	35 (44.9%)	14 (17.9%)	10 (12.8%)	19 (24.4%)
5-fu/fluorouracil	57	15 (26.3%)	13 (22.8%)	6 (10.5%)	23 (40.4%)
carboplatin	34	3 (8.8%)	11 (32.4%)	9 (26.5%)	11 (32.4%)
paclitaxel	25	10 (40%)	4 (16%)	3 (12%)	8 (32%)
**Cytotoxic antineoplastic classes**					
platinum agents	50	6 (12%)	18 (36%)	13 (26%)	13 (26%)
taxanes	32	12 (37.5%)	7 (21.9%)	3 (9.4%)	10 (31.2%)
**Non-cytotoxic antineoplastics**					
tamoxifen	30	7 (23.3%)	11 (36.7%)	3 (10%)	9 (30%)
asparaginase	25	3 (12%)	12 (48%)	1 (4%)	9 (36%)
**Kinase inhibitors**					
all kinase inhibitors	242*	116 (47.2%)	69 (28%)	23 (9.3%)	38 (15.4%)
imatinib	117	47 (40.2%)	26 (22.2%)	11 (9.4%)	33 (28.2%)
sunitinib	50	16 (32%)	16 (32%)	5 (10%)	13 (26%)
erlotinib	35	19 (54.3%)	6 (17.1%)	4 (11.4%)	6 (17.1%)
pazopanib	34	14 (41.2%)	11 (32.4%)	3 (8.8%)	6 (17.6%)
nilotinib	33	25 (75.8%)	3 (9.1%)	0 (0%)	5 (15.2%)
dasatinib	31	22 (71%)	4 (12.9%)	0 (0%)	5 (16.1%)
sorafenib	25	16 (64%)	2 (8%)	1 (4%)	6 (24%)
**Antineoplastic monoclonal antibodies**					
all monoclonal antibodies	39	16 (41%)	7 (17.9%)	2 (5.1%)	14 (35.9%)

*Four reports were in two of the categories indicated.

Relatively few reports referred to ‘immunoassay’ (n=29) compared to ‘chromatography’ (n=157), both terms first appearing in 1992 (**Supplemental Table S2**). The first report involving HPLC was published in 1993 (n=66). The first report referring to ‘mass spectrometry/MS’ was published in 1999 (n=126), and for ‘tandem mass spectrometry/MS/MS’ in 2004 (n=115); most reports referring to mass spectrometry concerned tandem mass spectrometry (91%). More recent techniques identified include Raman spectroscopy (first identified in 2015, n=14), ‘surface plasmon resonance/SPR’ (first identified in 2012, n= 5), ‘sensor’ or ‘biosensor’ (first identified in 2015, n=13 and 5, respectively), and ‘aptamer’ for this type of sensor (first identified in 2021, n=2).

Blood or plasma were the principal sampling matrices across reports. Alternative sampling matrices were referred to in few reports: ‘urine’, ‘saliva’ and ‘hair’ in 5, 4 and 2 reports, respectively (**Figure 3**; **Supplemental Table S2**). The term ‘intracellular’ first appeared in 2006 (n=10) and ‘peripheral blood mononuclear cell/PBMC’ in 2021 (n=2). Terms referring to alternative sampling methods included ‘dried blood spots/DBS’ (n=26) and ‘volumetric absorptive microsampling/VAMS’ (n=2), first reported in 2011 and 2019, respectively.

We selected terms referring to special populations, including extremes of age and obesity (**Supplemental Table S2**). The terms child (n=44 reports), pae/ediatric (n=33 reports), neonate (n=6 reports), and infant (n=4 reports) were first identified in reports from 1983, 2007, 2015 and 2016, respectively. The term ‘elderly’ first appeared in 1994 (10 reports), and ‘obese’ in 2002 (n=3 reports). The primary comorbidity evaluated was renal disease and its consequences. The terms ‘renal function’, ‘acute kidney injury/AKI’, ‘hae/emodialysis’ and ‘end stage renal disease’ first appeared in reports from 1980, 2009, 2009 and 2018, and in 11, 2, 8 and 2 reports, respectively.

Terms relating to pharmacometrics, modelling and simulation appeared throughout the sample and those relating to dose adaptation and decision support (‘Bayesian’, ‘forecast’, ‘decision support’) first appeared in the 2000s (**Supplemental Table S2**). Concepts such as ‘target concentration intervention’ and ‘PK/PD’, first appeared in 2012 and 2014, in 5 and 7 reports, respectively (**Supplemental Table S2**). Regarding terms with greater than 25 reports, ‘population pharmacokinetic’, ‘Bayesian’ and ‘simulation’ primarily featured in reports about modelling rather than other study categories. ‘Trough’ and ‘AUC’, however, appeared more frequently in clinical or other primary studies (**Supplemental Table S3**). Terms related to metabolism and pharmacogenetics/genomics appeared throughout the sample, with terms referring to metabolic enzymes (CYP, P450) first appearing in 2005 (**Supplemental Table S2**). The stem ‘pharmacogen-’ predominantly appeared in reviews and perspectives.

Other unique concepts that appeared throughout the sample included ‘toxic/toxicity’, ‘matrices/matrix’ and ‘targeted’. ‘Cost’ first appeared in 2010 (n=35). An emerging term of interest identified was ‘circadian’ (first report 2015, n=2). (**Supplemental Table S2**).

## Discussion

Pharmacological therapy is the mainstay of treatment for cancer patients. Interest in the application of TDM for dosing antineoplastics is suggested by the rise in publications that included this term in titles and abstracts over the last 10 years. This coincides with the advent of kinase inhibitors, where numerous diverse agents enter global markets annually. Advances in analytical instrumentation and sampling methodologies likely also play a role in the rise of such publications, and this is reflected in our timeline.

Of 27 cytotoxic antineoplastic agents among reports, four had 25 or more publications identified (methotrexate, 5-fluorouracil, carboplatin, paclitaxel; **Table 1**). Though not universal, TDM is established practice for two of these agents, methotrexate and 5-fluorouracil, while for paclitaxel evidence is emerging and clinical application is at present not widespread (2, 11, 12). Although taxanes were discovered in the 1960s, paclitaxel, the first commercially available agent, obtained regulatory approval in 1992 due to challenges with synthesis and formulation (13). It is thus reasonable that the first report about paclitaxel therapeutic drug monitoring we identified was published in 2000.

Carboplatin deserves special mention. About a third of studies concerning this drug were clinical and other primary studies. Dosing is determined by the Calvert formula which relates renal function to a target AUC (14). This approach does not require measuring drug concentrations and is therefore not an example of the application of TDM. However, the example represents a departure from BSA dosing that came from the evaluation of drug concentrations through clinical use that ultimately resulted in reduced interindividual variability and improved outcomes. Among clinical studies within our sample, drug monitoring for carboplatin was used for high dose chemotherapy (15), neonates and infants (16, 17), amputees (18), obesity (19), and determining irreversible alopecia (20); several of these were case reports. This suggests interest in the application of concentration measurement of carboplatin to optimise dosing in special populations and unique scenarios. Similarly, that 5-fluorouracil concentrations achieved by BSA dosing differ in women compared to men was determined by clinical pharmacokinetic studies, and this is now accounted for in BSA based formulae (21); dosing based on concentration measurement for 5-fluorouracil is nonetheless superior in reducing inter-patient variability in systemic concentrations.

Central to performing TDM is ready access to relevant assays that are precise and sensitive. Commercial immunoassays are available for a limited number of antineoplastics; methotrexate (globally), 5-fluorouracil, paclitaxel and docetaxel (Europe only) (2). Of the 29 reports we identified with the term ‘immunoassay’, 25 related to analytical aspects and 16 concerned methotrexate. While chromatographic assays have been developed for many agents, few have attained regulatory approval for clinical use (2). At present, most laboratories employ LC-MS/MS as the principal analytic method applied to measure these drugs, reflected by the large number of reports since 2004 in our sample (n=115), relative to U/HPLC and traditional mass spectrometry.

The availability of suitable instrumentation and methodology has likely impacted which drugs are evaluated as TDM candidates over time. Vincristine provides an example of an agent, that despite a long history of clinical use (13), first appeared in our sample in 2001. While an analytical method was published in 1985 (22), improved separation procedures and novel detection sensors resulted in more sensitive assays (23–25). These reports did not refer to therapeutic drug monitoring and thus did not appear in our sample. The earlier assays required relatively large sample volumes, and further procedural and instrumental improvements enabled concentration measurement in paediatric populations, especially neonates and children, as illustrated by three publications we identified (16, 26, 27).

Kinase inhibitors were referred to in 17.5% of all reports identified in our sample; most publications referred to analytical aspects (47.2%). Of 25 kinase inhibitors, seven had 25 or more publications identified (28%), and reports about analytical aspects comprised 32-76% of publications for individual agents. Kinase inhibitors are typically marketed at a single dose but exhibit substantial interindividual variability (6), and the impact of interindividual variability on achievement of suggested trough targets varies by agent. For imatinib and sunitinib, an estimated 73% and 49% of individuals fail to meet targets for efficacy with standardised dosing, while for erlotinib a majority achieve suggested targets (89%) (28).

TDM involves a multidisciplinary team, including clinical and laboratory staff, and represents a complex, service level intervention that involves multiple steps. Adoption of TDM by clinicians requires a change in the way they make dosing decisions. One consideration when exploring TDM in oncology is the need for practice change. Implementation of evidence-based approaches into clinical practice can take an average of 17 years (29). Avoiding this time lag necessitates identifying and addressing barriers to uptake. For TDM in other fields of medicine, such as infectious diseases, studies have identified barriers including time constraints, as well as integration of TDM processes into clinical workflow (30).

Perhaps key for incorporation of TDM in oncology, are barriers aligned with knowledge (30). As evidence to support TDM in oncology is largely provided by academic initiatives, the body of literature is affected by factors such as limited funding and time, and may be the reason for a paucity of clinical studies. Lack of robust evidence for an exposure-response relationship is often cited as a primary reason why TDM is not widely adopted in oncology, and, in particular, lack of randomized controlled trials (31). In our sample we found 6 interventional controlled trials, all conducted relatively recently (2011–2021) (12, 32–36). Interventional trials involving TDM are difficult to undertake as TDM is a complex intervention; some specific challenges include inability to blind clinicians and lack of uptake of TDM-based dose advice by prescribers (37, 38).

We observed increasing interest in the application of TDM to guide antineoplastic dosing in special populations, for example, those that might not have been included in phase 3 clinical trials. Terms related to the very young (pae/ediatric, neonate, infant) were identified from 2007 onwards. Similarly, the term obese was first identified in 2002, albeit in relatively few reports. Availability of context-specific knowledge would help build clinician trust in the ability of TDM to guide dosing decisions, supporting sustained uptake.

Robust evidence for clinical implementation requires studies that are of a high quality. Quality evaluation of the studies in our sample was out of scope. While there is a reporting guide for clinical pharmacokinetic studies (39), this is not often used (published 2015; 70 citations in 2022). Risk of bias tools applied in evidence summaries, such as systematic reviews and clinical guidelines, are used to appraise quality. There are tools to cover a variety of study designs (40, 41), however those currently available do not reflect the particular challenges involved in clinical pharmacokinetic studies and studies that evaluate TDM as an intervention.

We limited our search to reports with the term ‘therapeutic drug monitoring’ in the title and abstract. This is not a limitation *per se*, but rather reflects our objective to perform a rapid scoping exercise, executed in a limited time frame. We selected this term as we consider it the most precise term to retrieve relevant reports. We excluded more general terms such as ‘drug monitoring’, ‘trough concentrations’ and ‘pharmacokinetics’ as these increased the retrieval of irrelevant reports many-fold. Our results must therefore be interpreted as reflecting the sample, rather than the complete body of work.

Work prior to 1990 might be underrepresented due to factors including increasing but not established adoption of the term, and relative lack of completeness of bibliographic databases prior to this date. The first report we captured is a review about methotrexate TDM and refers to previously published work that was not captured in our rapid scoping approach (42). Over time, reporting standards have developed, and it is possible that abstracts from earlier publications may have lacked detail compared to more recent work. Nevertheless, most antineoplastics currently in use were marketed after 1990, including some classic agents, such as paclitaxel, and all hormonal and targeted agents (13).

Concept based exploration was limited to concepts that could be defined by specific terms. For example, our approach did not permit determining changes in opinion about BSA based dosing over time, as it would be difficult to reduce this discourse to a set of key terms. Concept-based exploration was limited to the terms we proposed or identified through the tagging process. Topic modelling might be a helpful approach for future work (43), however might not capture important concepts of interest with low representation. Finally, the exploratory approach means that some agents not known to the authors with low representation in the data set may have been missed, however it is unlikely that highly represented agents were missed in the sample.

## Conclusions

We undertook a bibliometric evaluation of the literature referring to the TDM of antineoplastic agents. Our sample primarily concerned reports about analytical methods, and relatively few reports relating to clinical outcomes of a design to support implementation. Gaps related to the agents evaluated might be explained by instrumental developments, for example, LC-MS/MS enabling measurement of vincristine. TDM offers an opportunity to improve the effectiveness and safety of antineoplastics, particularly with complex drug regimes, high risk populations and perhaps even in resistant disease. However, more robust evidence is needed to support implementation.

## Data availability statement

The data that support the findings of this study are available on request from the corresponding author.

## Author contributions

JSt, JC, BM, and VS performed screening and tagging. JSt, JC, and BM analyzed and interpreted data. JSi and FL provided clinical feedback and discussion. JSt, JC, BM, and FL wrote the manuscript draft. JSt conceived and supervised the project. All authors contributed to the article and approved the submitted version.

## Funding

The article processing charge was supported by the Research Directorate of Universidad de Valparaíso (Grant CIDI8 Interdisciplinary Centre for Health Studies).

## Acknowledgments

The authors would like to acknowledge Christian Valero Berretta for preparing data summaries in R.

## Conflict of interest

The authors declare that the research was conducted in the absence of any commercial or financial relationships that could be construed as a potential conflict of interest.

## Publisher’s note

All claims expressed in this article are solely those of the authors and do not necessarily represent those of their affiliated organizations, or those of the publisher, the editors and the reviewers. Any product that may be evaluated in this article, or claim that may be made by its manufacturer, is not guaranteed or endorsed by the publisher.

## References

[1] Dasgupta A. Chapter 1 - introduction to therapeutic drug monitoring: Frequently and less frequently monitored drugs. In: Dasgupta A, editor. Therapeutic drug monitoring. Boston: Academic Press (2012). p. 1–29. Available at: https://www.sciencedirect.com/science/article/pii/B9780123854674000014.

[2] Knezevic CE Clarke W. Cancer chemotherapy: The case for therapeutic drug monitoring. Ther Drug Monit (2020) 42(1):6–19. doi: 10.1097/FTD.0000000000000701 31568180

[3] Fleisher B Ait-Oudhia S. A retrospective examination of the US food and drug administration’s clinical pharmacology reviews of oncology biologics for potential use of therapeutic drug monitoring. OncoTargets Ther (2018) 11:113–21. doi: 10.2147/OTT.S153056 PMC5749565 29343970

[4] Bayer plc. Stivarga 40 mg film-coated tablets - summary of product characteristics (SmPC). In: Electronic medicines compendium (emc) (2022). Available at: https://www.medicines.org.uk/emc/product/1263.

[5] Janssen-Cilag Ltd. Imbruvica 140 mg film-coated tablets - summary of product characteristics (SmPC). In: Electronic medicines compendium (emc) (2022). Available at: https://www.medicines.org.uk/emc/product/10025/smpc.

[6] Lucas CJ Martin JH. Pharmacokinetic-guided dosing of new oral cancer agents. J Clin Pharmacol (2017) 57(Suppl 10):S78–98. doi: 10.1002/jcph.937 28921641

[7] Widmer N Bardin C Chatelut E Paci A Beijnen J Levêque D. Review of therapeutic drug monitoring of anticancer drugs part two–targeted therapies. Eur J Cancer Oxf Engl 1990 (2014) 50(12):2020–36. doi: 10.1016/j.ejca.2014.04.015 24928190

[8] Feng Y Roy A Masson E Chen TT Humphrey R Weber JS. Exposure-response relationships of the efficacy and safety of ipilimumab in patients with advanced melanoma. Clin Cancer Res Off J Am Assoc Cancer Res (2013) 19(14):3977–86. doi: 10.1158/1078-0432.CCR-12-3243 23741070

[9] Ouzzani M Hammady H Fedorowicz Z Elmagarmid A. Rayyan–a web and mobile app for systematic reviews. Syst Rev (2016) 5(1):1–10. doi: 10.1186/s13643-016-0384-4 27919275 PMC5139140

[10] R Core Team. R: A language and environment for statistical computing. In: Vienna, Austria: R foundation for statistical computing (2022). Available at: https://www.R-project.org/.

[11] Muth M Ojara FW Kloft C Joerger M. Role of TDM-based dose adjustments for taxane anticancer drugs. Br J Clin Pharmacol (2021) 87(2):306–16. doi: 10.1111/bcp.14678 33247980

[12] Joerger M von Pawel J Kraff S Fischer JR Eberhardt W Gauler TC. Open-label, randomized study of individualized, pharmacokinetically (PK)-guided dosing of paclitaxel combined with carboplatin or cisplatin in patients with advanced non-small-cell lung cancer (NSCLC). Ann Oncol Off J Eur Soc Med Oncol (2016) 27(10):1895–902. doi: 10.1093/annonc/mdw290 27502710

[13] Chabner BA Roberts TG. Chemotherapy and the war on cancer. Nat Rev Cancer (2005) 5(1):65–72. doi: 10.1038/nrc1529 15630416

[14] Calvert AH Newell DR Gumbrell LA O’Reilly S Burnell M Boxall FE. Carboplatin dosage: Prospective evaluation of a simple formula based on renal function. J Clin Oncol Off J Am Soc Clin Oncol (1989) 7(11):1748–56. doi: 10.1200/JCO.1989.7.11.1748 2681557

[15] Chevreau C Massard C Flechon A Delva R Gravis G Lotz JP. Multicentric phase II trial of TI-CE high-dose chemotherapy with therapeutic drug monitoring of carboplatin in patients with relapsed advanced germ cell tumors. Cancer Med (2021) 10(7):2250–8. doi: 10.1002/cam4.3687 PMC7982623 33675184

[16] Veal GJ Errington J Sastry J Chisholm J Brock P Morgenstern D. Adaptive dosing of anticancer drugs in neonates: Facilitating evidence-based dosing regimens. Cancer Chemother Pharmacol (2016) 77(4):685–92. doi: 10.1007/s00280-016-2975-0 PMC4819938 26875154

[17] Nijstad AL van Eijkelenburg NKA Kraal KCJM Meijs MJM de Kanter CTMM Lilien MR. Cisplatin and carboplatin pharmacokinetics in a pediatric patient with hepatoblastoma receiving peritoneal dialysis. Cancer Chemother Pharmacol (2020) 86(3):445–9. doi: 10.1007/s00280-020-04130-z PMC7479000 32816154

[18] van Gorp F van Rens MTM Kuck EM Rozemeijer R Huitema ADR Brouwers EEM. Dosing of carboplatin in a patient with amputated legs: A case report. J Oncol Pharm Pract Off Publ Int Soc Oncol Pharm Pract (2014) 20(6):473–5. doi: 10.1177/1078155213514470 24356803

[19] De Jonge ME Mathôt RAA Van Dam SM Beijnen JH Rodenhuis S. Extremely high exposures in an obese patient receiving high-dose cyclophosphamide, thiotepa and carboplatin. Cancer Chemother Pharmacol (2002) 50(3):251–5. doi: 10.1007/s00280-002-0494-7 12203108

[20] de Jonge ME Mathôt R a. A Dalesio O Huitema ADR Rodenhuis S Beijnen JH. Relationship between irreversible alopecia and exposure to cyclophosphamide, thiotepa and carboplatin (CTC) in high-dose chemotherapy. Bone Marrow Transplant (2002) 30(9):593–7. doi: 10.1038/sj.bmt.1703695 12407434

[21] Mueller F Büchel B Köberle D Schürch S Pfister B Krähenbühl S. Gender-specific elimination of continuous-infusional 5-fluorouracil in patients with gastrointestinal malignancies: Results from a prospective population pharmacokinetic study. Cancer Chemother Pharmacol (2013) 71(2):361–70. doi: 10.1007/s00280-012-2018-4 23139054

[22] De Smet M Van Belle SJ Storme GA Massart DL. High-performance liquid chromatographic determination of vinca-alkaloids in plasma and urine. J Chromatogr (1985) 345(2):309–21. doi: 10.1016/0378-4347(85)80168-7 4086600

[23] Bloemhof H Van Dijk KN De Graaf SS Vendrig DE Uges DR. Sensitive method for the determination of vincristine in human serum by high-performance liquid chromatography after on-line column-extraction. J Chromatogr (1991) 572(1–2):171–9. doi: 10.1016/0378-4347(91)80481-Q 1818052

[24] Crom WR de Graaf SS Synold T Uges DR Bloemhof H Rivera G. Pharmacokinetics of vincristine in children and adolescents with acute lymphocytic leukemia. J Pediatr (1994) 125(4):642–9. doi: 10.1016/S0022-3476(94)70027-3 7931891

[25] de Graaf SS Bloemhof H Vendrig DE Uges DR. Vincristine disposition in children with acute lymphoblastic leukemia. Med Pediatr Oncol (1995) 24(4):235–40. doi: 10.1002/mpo.2950240405 7700168

[26] Koopmans P Gidding CE de Graaf SS Uges DR. An automated method for the bioanalysis of vincristine suitable for therapeutic drug monitoring and pharmacokinetic studies in young children. Ther Drug Monit (2001) 23(4):406–9. doi: 10.1097/00007691-200108000-00014 11477324

[27] Corona G Casetta B Sandron S Vaccher E Toffoli G. Rapid and sensitive analysis of vincristine in human plasma using on-line extraction combined with liquid chromatography/tandem mass spectrometry. Rapid Commun Mass Spectrom RCM (2008) 22(4):519–25. doi: 10.1002/rcm.3390 18228243

[28] Lankheet NAG Knapen LM Schellens JHM Beijnen JH Steeghs N Huitema ADR. Plasma concentrations of tyrosine kinase inhibitors imatinib, erlotinib, and sunitinib in routine clinical outpatient cancer care. Ther Drug Monit (2014) 36(3):326–34. doi: 10.1097/FTD.0000000000000004 24305627

[29] Morris ZS Wooding S Grant J. The answer is 17 years, what is the question: understanding time lags in translational research. J R Soc Med (2011) 104(12):510–20. doi: 10.1258/jrsm.2011.110180 PMC3241518 22179294

[30] Chan JOS Baysari MT Carland JE Sandaradura I Moran M Day RO. Barriers and facilitators of appropriate vancomycin use: Prescribing context is key. Eur J Clin Pharmacol (2018) 74(11):1523–9. doi: 10.1007/s00228-018-2525-2 30056569

[31] Saleem M Dimeski G Kirkpatrick CM Taylor PJ Martin JH. Target concentration intervention in oncology: where are we at? Ther Drug Monit (2012) 34(3):257–65. doi: 10.1097/FTD.0b013e3182557342 22585183

[32] Engels FK Loos WJ van der Bol JM de Bruijn P Mathijssen RHJ Verweij J. Therapeutic drug monitoring for the individualization of docetaxel dosing: a randomized pharmacokinetic study. Clin Cancer Res Off J Am Assoc Cancer Res0 (2011) 17(2):353–62. doi: 10.1158/1078-0432.CCR-10-1636 21224369

[33] Gotta V Widmer N Decosterd LA Chalandon Y Heim D Gregor M. Clinical usefulness of therapeutic concentration monitoring for imatinib dosage individualization: Results from a randomized controlled trial. Cancer Chemother Pharmacol (2014) 74(6):1307–19. doi: 10.1007/s00280-014-2599-1 25297989

[34] de Wit D van Erp NP den Hartigh J Wolterbeek R den Hollander-van Deursen M Labots M. Therapeutic drug monitoring to individualize the dosing of pazopanib: A pharmacokinetic feasibility study. Ther Drug Monit (2015) 37(3):331–8. doi: 10.1097/FTD.0000000000000141 25271729

[35] Zhang J Zhou F Qi H Ni H Hu Q Zhou C. Randomized study of individualized pharmacokinetically-guided dosing of paclitaxel compared with body-surface area dosing in Chinese patients with advanced non-small cell lung cancer. Br J Clin Pharmacol (2019) 85(10):2292–301. doi: 10.1111/bcp.13982 PMC6783603 31077432

[36] Rousselot P Mollica L Guilhot J Guerci A Nicolini FE Etienne G. Dasatinib dose optimisation based on therapeutic drug monitoring reduces pleural effusion rates in chronic myeloid leukaemia patients. Br J Haematol (2021) 194(2):393–402. doi: 10.1111/bjh.17654 34195988

[37] Kredo T der WJSV Siegfried N Cohen K. Therapeutic drug monitoring of antiretrovirals for people with HIV. Cochrane Database of Systematic Reviews (2009) (3). doi: 10.1002/14651858.CD007268.pub2 19588422

[38] Scheetz MH Lodise TP Downes KJ Drusano G Neely M. The case for precision dosing: medical conservatism does not justify inaction. J Antimicrob Chemother (2021) 76(7):1661–5. doi: 10.1093/jac/dkab086 PMC8212772 33843994

[39] Kanji S Hayes M Ling A Shamseer L Chant C Edwards DJ. Reporting guidelines for clinical pharmacokinetic studies: The ClinPK statement. Clin Pharmacokinet (2015) 54(7):783–95. doi: 10.1007/s40262-015-0236-8 25637173

[40] Higgins JPT Savović J Page MJ Elbers RG Sterne JAC. Chapter 8: Assessing risk of bias in a randomized trial. In: Higgins JPT Thomas J Chandler J Cumpston M Li T Page MJ. (editors). Cochrane Handbook for Systematic Reviews of Interventions version 6.3 (updated February 2022). Cochrane (2022). Available from: www.training.cochrane.org/handbook.

[41] Sterne JA Hernán MA McAleenan A Reeves BC Higgins JP. Chapter 25: Assessing risk of bias in a non-randomized study. In: Higgins JPT Thomas J Chandler J Cumpston M Li T Page MJ. (editors). Cochrane Handbook for Systematic Reviews of Interventions version 6.3 (updated February 2022). Cochrane (2022). Available from: https://training.cochrane.org/handbook/current/chapter-25.

[42] Sadée W. Antineoplastic agents: high-dose methotrexate and citrovorum factor rescue. Ther Drug Monit (1980) 2(2):177–85. doi: 10.1097/00007691-198004000-00012 6762711

[43] Asmussen CB Møller C. Smart literature review: A practical topic modelling approach to exploratory literature review. J Big Data (2019) 6(1):93. doi: 10.1186/s40537-019-0255-7

