# Supplementary material for: Therapeutic drug monitoring in oncology - What’s out there: A bibliometric evaluation on the topic
Source: Front Oncol. 2022 Nov 10;12:959741. doi: 10.3389/fonc.2022.959741 (PMC9685987; doi:10.3389/fonc.2022.959741)
Supplement: Supplementary file 1 [file DataSheet_1.docx]

Supplementary Material

1. Appendices
2. Supplemental Data

# Appendices

**Appendix 1 – Terms used to separate studies related to oncology/haematology**

| **General terms in oncology/haematology:** |
| --- |
| cancer, oncolog, hematolo, haematol, neoplas, carcinoma, malignan, metasta, tumour, tumor, chemotherapy, cytotoxic |
| **Terms related to common antineoplastic agents:** |
| *Cytotoxic antineoplastics***:** methotrexate, busulfan, fluorouracil, 5-fu, capecitabine, mercaptopurine, taxel, platin, cyclophosphamide, asparaginase, alidomide, etoposide  *Hormonal antineoplastics:* tamoxifen, abiraterone, mitotane, hormonal, lutamide, androgen  *Kinase inhibitors:* tyrosine, tinib, fenib, clib, inib, anib  *Monoclonal antibodies:* mab |

**Appendix 2 – Study design tagging**

| **Study design** | **Publication type category** |
| --- | --- |
| assay development and/or validation, novel extraction method, alternative sampling method | Analytical method development and validation |
| case report, case series, prospective cohorts, retrospective cohorts, randomised controlled trials/non-randomised controlled trials, cost effectiveness analyses | Clinical trials and primary studies |
| narrative review, systematic review, systematic review with meta-analysis, editorial, letter, comment, perspective, guideline, protocol (n=1) | Reviews and perspectives |
| population pharmacokinetic modelling, Monte Carlo simulation | Modelling and simulation |

1. **Supplemental data**

**A complete, tagged data file of our sample can be accessed at:** <https://osf.io/t2yvg>

**Supplemental Table S1. All antineoplastics: frequency and year of first report**

|  | **Frequency** | **Year of first report** |
| --- | --- | --- |
| **1. Cytotoxic antineoplastics** | | |
| **1.1 Alkylating agents** | 36 | 1986 |
| busulfan | 20 | 1993 |
| cyclophosphamide | 16 | 1986 |
| **1.2 Anthracyclines** | 19 | 1993 |
| doxorubicin | 14 | 1993 |
| daunorubicin | 3 | 1999 |
| amrubicin | 1 | 2005 |
| epirubicin | 1 | 1996 |
| **1.3 Antimetabolites** | 174 | 1980 |
| methotrexate | 76 | 1980 |
| fluorouracil | 56 | 1990 |
| mercaptopurine | 16 | 1986 |
| capecitabine | 9 | 2003 |
| thioguanine (tioguanine n=0) | 6 | 2009 |
| gemcitabine | 5 | 2006 |
| cytarabine/cytosine arabinoside | 4 | 1986 |
| pemetrexed | 2 | 2016 |
| **1.4 Platinum compounds** | 57 | 1992 |
| carboplatin | 34 | 1992 |
| cisplatin | 16 | 1993 |
| oxaliplatin | 7 | 2006 |
| **1.5 Taxanes** | 41 | 2000 |
| paclitaxel | 25 | 2000 |
| docetaxel | 16 | 2009 |
| **1.6 Topoisomerase I inhibitors** | 21 | 1999 |
| irinotecan | 20 | 1999 |
| topotecan | 1 | 2002 |
| **1.7 Vinca alkaloid** | 8 | 2001 |
| vinblastine | 3 | 2008 |
| vincristine | 3 | 2001 |
| vinorelbine | 2 | 2018 |
| **1.8 Other cytotoxic antineoplastics** | 28 | 1987 |
| etoposide | 24 | 1990 |
| bleomycin | 4 | 1987 |
| **2. Noncytotoxic antineoplastics** | | |
| **2.1 Hormonal antineoplastics** | **83** | **2004** |
| tamoxifen | 30 | 2004 |
| abiraterone | 15 | 2015 |
| mitotane | 10 | 2014 |
| enzalutamide | 7 | 2017 |
| anastrozole | 4 | 2016 |
| letrozole | 4 | 2016 |
| exemestane | 3 | 2019 |
| bromocriptine | 1 | 2015 |
| octreotide | 1 | 2014 |
| **2.2 Antineoplastic antibodies** | **44** | **2009** |
| bevacizumab | 13 | 2010 |
| cetuximab | 9 | 2009 |
| nivolumab | 7 | 2018 |
| pembrolizumab | 7 | 2018 |
| rituximab | 6 | 2012 |
| panitumumab | 1 | 2018 |
| ramucirumab | 1 | 2021 |
| **2.3 Kinase inhibitors** | **500*** | **2005** |
| imatinib | 116 | 2005 |
| sunitinib | 49 | 2009 |
| erlotinib | 34 | 2009 |
| pazopanib | 34 | 2012 |
| nilotinib | 33 | 2009 |
| dasatinib | 31 | 2009 |
| sorafenib | 25 | 2009 |
| apatinib | 18 | 2009 |
| lapatinib | 16 | 2009 |
| gefitinib | 15 | 2009 |
| dabrafenib | 14 | 2016 |
| vemurafenib | 13 | 2014 |
| afatinib | 12 | 2015 |
| bosutinib | 12 | 2012 |
| ponatinib | 12 | 2017 |
| ibrutinib | 11 | 2015 |
| trametinib | 11 | 2016 |
| crizotinib | 9 | 2015 |
| axitinib | 8 | 2011 |
| regorafenib | 8 | 2018 |
| osimertinib | 7 | 2016 |
| cabozantinib | 4 | 2020 |
| lenvatinib | 4 | 2019 |
| anlotinib | 2 | 2021 |
| palbociclib | 2 | 2020 |
| **2.4 Other non-cytotoxic antineoplastic agents** | 27 | 2002 |
| asparaginase | 25 | 2002 |
| lenalidomide | 1 | 2016 |
| thalidomide | 1 | 2006 |

** A single publication may have referred to multiple kinase inhibitors*

**Supplemental Table S2. Terms for emerging topics and technologies, frequency and year of first report**

|  | **Frequency** | **Year of first report** |
| --- | --- | --- |
| **1 Analytical techniques** | |  |
| **1.1 Chromatography** | |  |
| liquid chromatography | 146 | 1992 |
| high performance | 49 | 1992 |
| HPLC | 66 | 1992 |
| UHPLC | 8 | 2014 |
| **1.2 Immunoassay** | |  |
| immunoassay | 29 | 1992 |
| EMIT | 3 | 1996 |
| kit | 12 | 2008 |
| Enzyme linked immunosorbent assay | 9 | 2009 |
| ELISA | 18 | 2011 |
| **1.3 Mass Spectrometry** | |  |
| mass spectrometry | 126 | 1999 |
| MS-MS | 115 | 2004 |
| tandem mass | 105 | 2004 |
| LC-MS-MS | 101 | 2008 |
| **1.4 Raman Spectroscopy** | | |
| SERS | 14 | 2015 |
| raman | 10 | 2016 |
| surface enhanced raman spectroscopy | 3 | 2016 |
| **1.5 Biosensor** | |  |
| plasmon | 7 | 2012 |
| surface plasmon resonance | 5 | 2012 |
| biosensor | 5 | 2015 |
| sensor | 13 | 2015 |
| **1.6 Detection** | |  |
| fluorescent | 6 | 1995 |
| ultrasensitive | 2 | 2016 |
| aptamer | 2 | 2021 |
| **1.7 Ionization** | |  |
| desorption | 4 | 2010 |
| paper.spray | 7 | 2011 |
| **1.8 Extraction** | |  |
| extraction | 89 | 1993 |
| solid phase extraction | 12 | 2004 |
| **1.9 Separation** | |  |
| nanopillar assisted separation | 1 | 2021 |
| **2.2 Sampling** | |  |
| urine | 21 | 1980 |
| saliva | 4 | 1992 |
| hair | 2 | 2002 |
| intracellular | 10 | 2006 |
| PBMC | 2 | 2021 |
| dried blood spot/DBS | 26 | 2011 |
| microsample | 1 | 2019 |
| volumetric absorptive microsampling | 2 | 2019 |
| alternative sampling | 1 | 2021 |
| **2.3 Special patient groups** | | |
| neonate | 6 | 2015 |
| infant | 4 | 2016 |
| child | 44 | 1983 |
| pediatric | 28 | 2007 |
| paediatric | 5 | 2010 |
| adult | 27 | 1994 |
| elderly | 10 | 1994 |
| older | 10 | 1994 |
| older patient | 4 | 2017 |
| renal function | 11 | 1980 |
| acute kidney | 2 | 2009 |
| end stage renal disease | 2 | 2018 |
| hemodialysis | 7 | 2009 |
| haemodialysis | 1 | 2021 |
| obese | 3 | 2002 |
| **2.4 Pharmacometrics, modelling and simulation** | | |
| AUC | 60 | 1989 |
| population pharmacokinetic | 39 | 1996 |
| poppk | 3 | 2017 |
| trough | 92 | 1998 |
| simulation | 26 | 2000 |
| Bayesian | 34 | 2001 |
| forecast | 4 | 2003 |
| decision.support | 3 | 2008 |
| decision_1 | 25 | 2008 |
| decision_2 | 10 | 2008 |
| target concentration intervention | 5 | 2012 |
| TCI | 3 | 2012 |
| PK/PD | 7 | 2014 |
| pharmacometric | 3 | 2019 |
| **2.5 Metabolism and pharmacogenetics/genomics** | | |
| interaction | 53 | 1987 |
| metabolite | 152 | 1992 |
| genotype | 25 | 1999 |
| pharmacogen | 32 | 1999 |
| CYP | 27 | 2005 |
| P450 | 13 | 2005 |
| pgx | 1 | 2020 |
| **2.6 Unique concepts** | |  |
| toxic | 240 | 1980 |
| toxicity | 179 | 1980 |
| matrices | 6 | 1997 |
| matrix | 61 | 1997 |
| targeted | 56 | 1998 |
| novel | 22 | 2001 |
| cost | 35 | 2010 |
| nano | 26 | 2012 |
| circadian | 2 | 2015 |
| PD-1 | 4 | 2018 |
| CTLA-4 | 3 | 2019 |
| checkpoint | 7 | 2019 |

**Supplemental Table S3. Publication type identified for the most commonly reported concepts across the sample (25 reports or more).**

| **CONCEPT** | **Total** | **Analytical method development and validation** | **Clinical trials and primary studies** | **Modelling and simulation** | **Reviews and perspectives** |
| --- | --- | --- | --- | --- | --- |
| **Analytical Method** |  |  |  |  |  |
| Liquid chromatography | 149 | 121 (81.2%) | 17 (11.4%) | 4 (2.7%) | 7 (4.7%) |
| mass spectrometry | 129 | 111 (86%) | 11 (8.5%) | 1 (0.8%) | 6 (4.7%) |
| MS-MS | 115 | 108 (93.9%) | 6 (5.2%) | NA (NA%) | 1 (0.9%) |
| tandem mass | 108 | 95 (88%) | 9 (8.3%) | 1 (0.9%) | 3 (2.8%) |
| LC-MS-MS | 101 | 95 (94.1%) | 5 (5%) | NA (NA%) | 1 (1%) |
| HPLC (includes UHPLC) | 67 | 56 (83.6%) | 4 (6%) | 4 (6%) | 3 (4.5%) |
| high performance | 52 | 38 (73.1%) | 10 (19.2%) | 3 (5.8%) | 1 (1.9%) |
| immunoassay | 30 | 25 (83.3%) | 4 (13.3%) | 1 (3.3%) | NA (NA%) |
| **Special Populations** |  |  |  |  |  |
| child | 45 | 7 (15.6%) | 19 (42.2%) | 7 (15.6%) | 12 (26.7%) |
| pediatric | 28 | 4 (14.3%) | 11 (39.3%) | 5 (17.9%) | 8 (28.6%) |
| adult | 27 | 5 (18.5%) | 6 (22.2%) | 6 (22.2%) | 10 (37%) |
| **Pharmacometrics** |  |  |  |  |  |
| trough | 92 | 17 (18.5%) | 50 (54.3%) | 10 (10.9%) | 15 (16.3%) |
| AUC | 60 | 4 (6.7%) | 28 (46.7%) | 19 (31.7%) | 9 (15%) |
| population pharmacokinetic | 40 | 4 (10%) | 2 (5%) | 29 (72.5%) | 5 (12.5%) |
| Bayesian | 35 | 2 (5.7%) | 8 (22.9%) | 15 (42.9%) | 10 (28.6%) |
| simulation | 29 | 3 (10.3%) | 3 (10.3%) | 19 (65.5%) | 4 (13.8%) |
| decision | 25 | 7 (28%) | 6 (24%) | 3 (12%) | 9 (36%) |
| **Pharmacogenetics and metabolism** |  |  |  |  |  |
| metabolite | 154 | 86 (55.8%) | 27 (17.5%) | 19 (12.3%) | 22 (14.3%) |
| interaction | 54 | 17 (31.5%) | 14 (25.9%) | 6 (11.1%) | 17 (31.5%) |
| pharmacogen' | 33 | 2 (6.1%) | 6 (18.2%) | NA (NA%) | 25 (75.8%) |
| CYP | 27 | 3 (11.1%) | 10 (37%) | 5 (18.5%) | 9 (33.3%) |
| genotype | 25 | 4 (16%) | 6 (24%) | 4 (16%) | 11 (44%) |
| **Other terms** |  |  |  |  |  |
| targeted | 56 | 12 (21.4%) | 8 (14.3%) | 5 (8.9%) | 31 (55.4%) |
| cost | 35 | 14 (40%) | 12 (34.3%) | 1 (2.9%) | 8 (22.9%) |
| dried blood spot/DBS | 27 | 24 (88.9%) | 1 (3.7%) | NA (NA%) | 2 (7.4%) |
| nano | 26 | 18 (69.2%) | 1 (3.8%) | NA (NA%) | 7 (26.9%) |
